# Supplementary figures and images for: Nuclear p62/SQSTM1 facilitates ubiquitin-independent proteasomal degradation of BMAL1
Source: PLoS Genet. 2025 Jul 10;21(7):e1011794. doi: 10.1371/journal.pgen.1011794 (PMC12266388; doi:10.1371/journal.pgen.1011794)

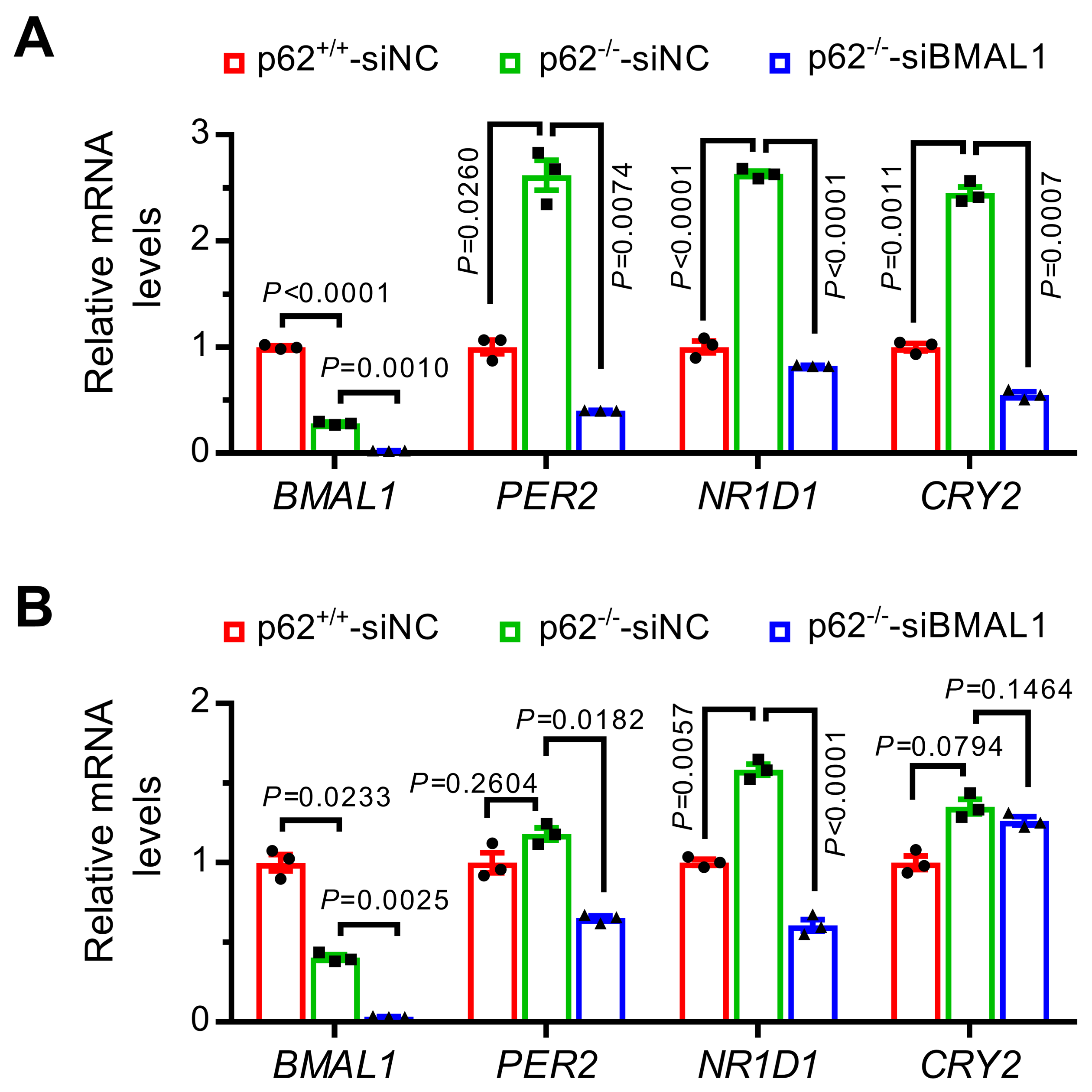

Supplement: S1 Fig — p62+/+ and p62-/- cells were transfected with indicated siRNAs. After 48 h, the cells were treated with Dexamethasone (100 μM) for 2 h. The mRNA levels of clock genes were then analysis at 4 h (A) and 16 h (B). Data are mean ± SEM of biological replicates (n = 3). The P value was determined by a one-way ANOVA analysis. (TIF) [file pgen.1011794.s001.tif]

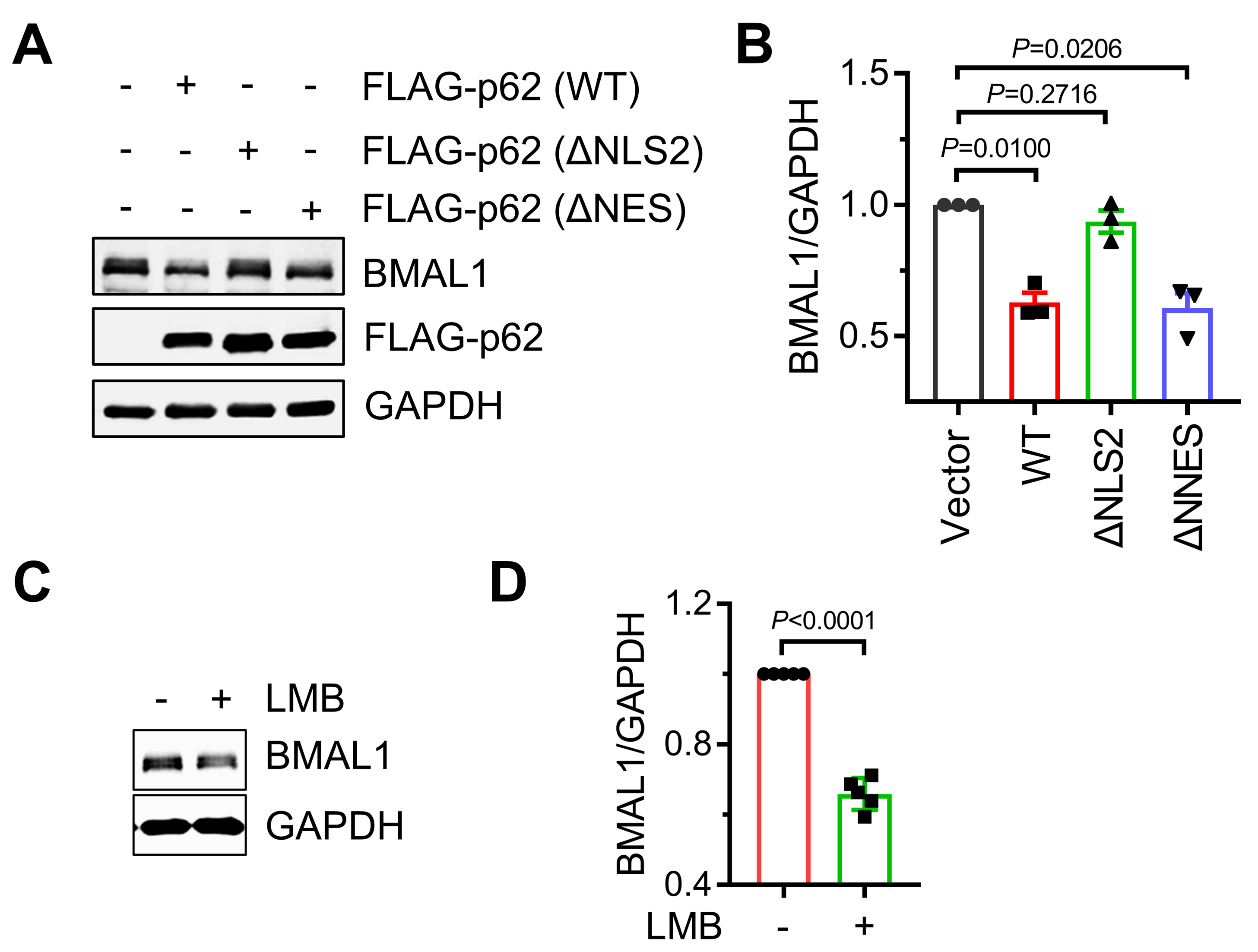

Supplement: S2 Fig — (A) p62 knock out AD293 (p62-/-) cells were transfected with the indicated plasmids. After 24 h, the cells were lysed and subjected to western blot analysis with indicated antibodies. (B) Quantitative analysis of results in (A). Data are mean ± SEM of biological replicates (n = 3). (C) HEK293 cells were treated with LMB (2 μM)) for 16 h. The whole-cell lysates were subjected to western blot analysis with indicated antibodies. (D) Quantitative analysis of results in (C). Data are mean ± SEM of biological replicates (n = 3). For B and D, the P value was determined by Student’s t test (two-sided). (TIF) [file pgen.1011794.s002.tif]

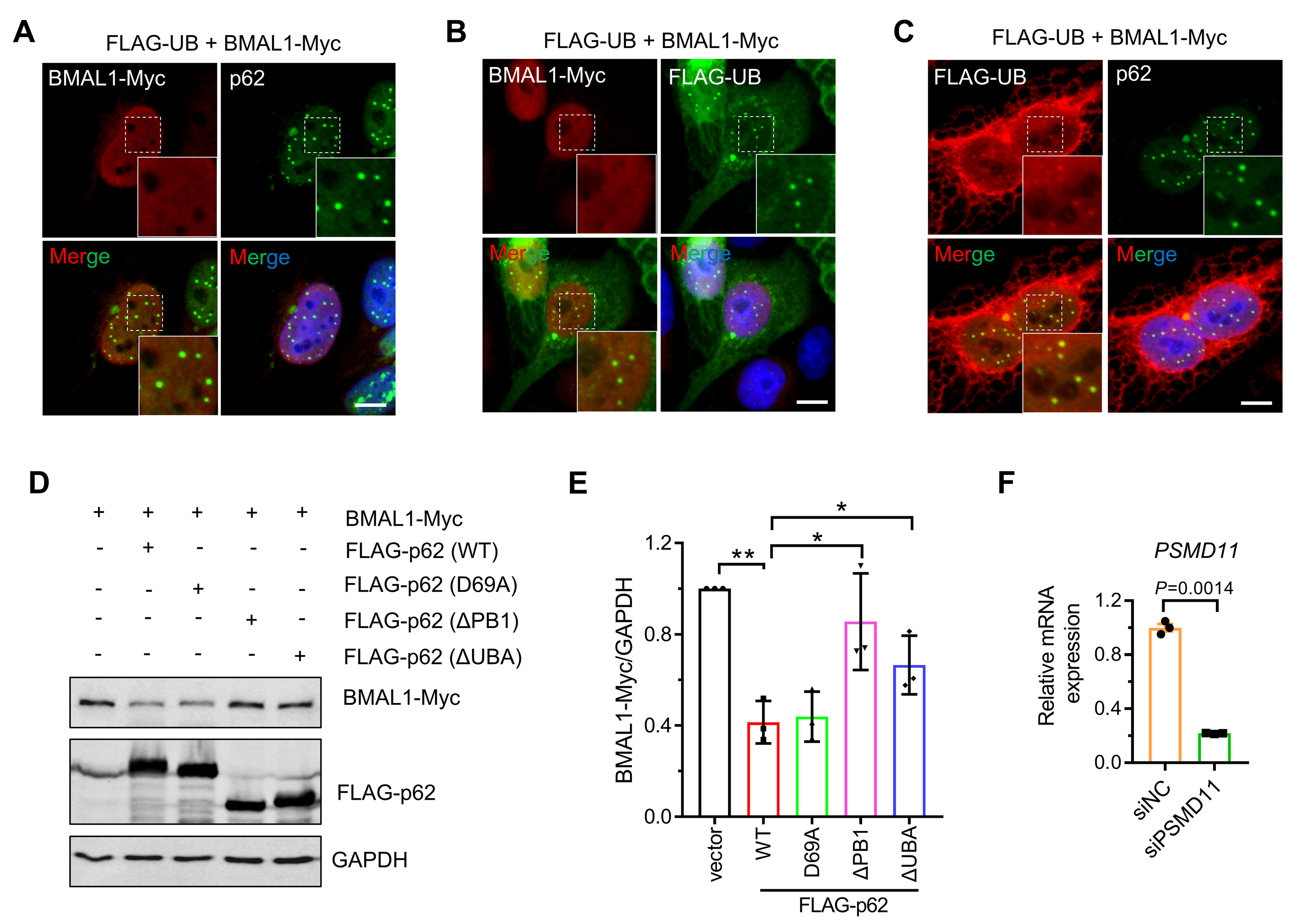

Supplement: S3 Fig — (A-C) HeLa cells were transfected with FLAG-ubiquitin (FLAG-UB) and BMAL1-Myc, then treated with MG132 (2 μM) and LMB (2 μM) for 16 hours. Cells were subsequently immunostained with the indicated antibodies. Nuclei were stained with DAPI (blue). Scale bar: 10 μm. (D) p62 knock out AD293 (p62-/-) cells were transfected with the indicated plasmids. After 24 h, the cells were lysed and subjected to western blot analysis with indicated antibodies. (E) Quantitative analysis of results in (D). Data are mean ± SEM of biological replicates (n = 3). (F) HEK293 cells were transfected with indicated siRNA for 48 h, the mRNA levels of PSMD11 were analyzed by RT-qPCR. Data are mean ± SEM of biological replicates (n = 3). For E and F, the P value was determined by Student’s t test (two-sided). (TIF) [file pgen.1011794.s003.tif]

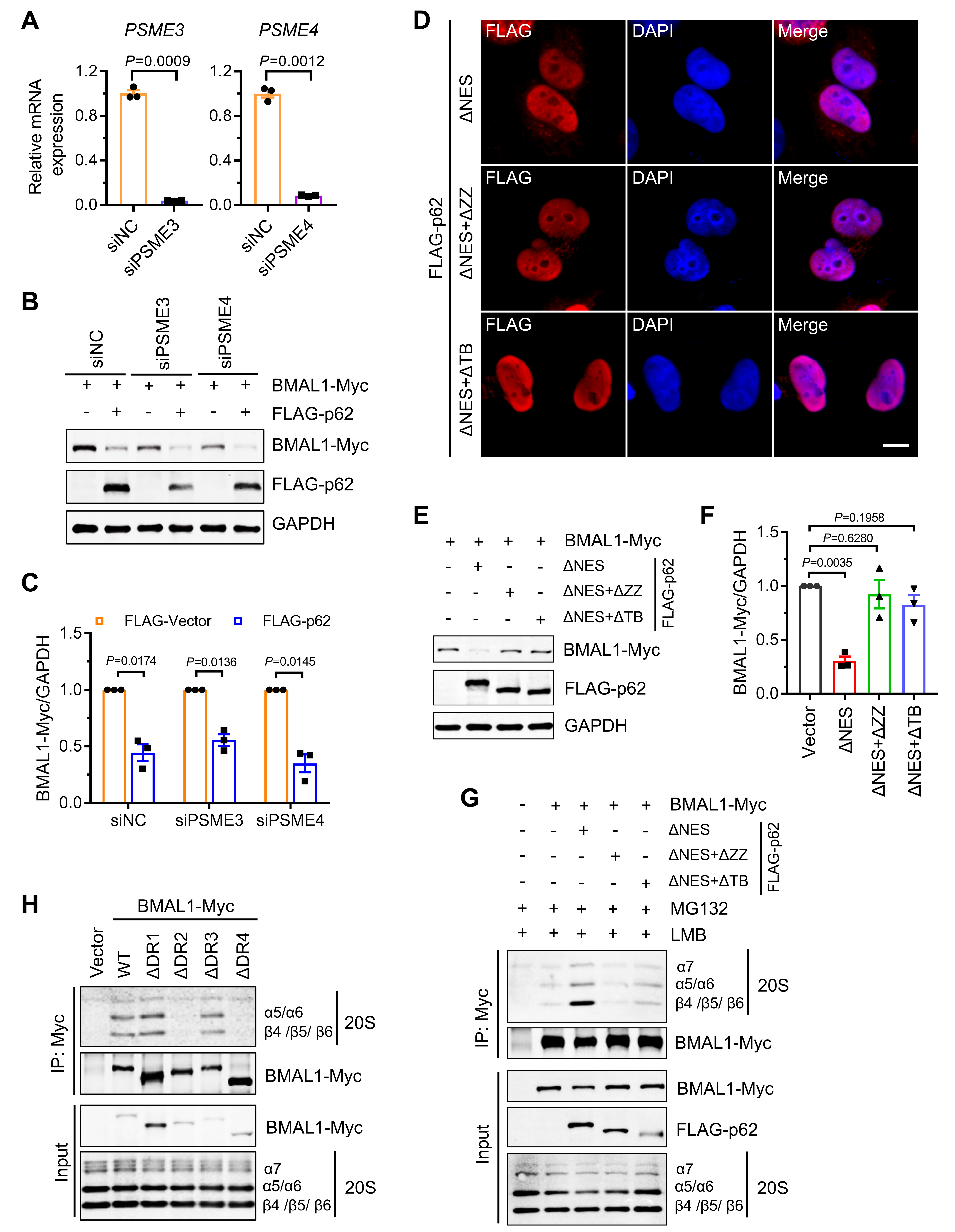

Supplement: S4 Fig — (A) HEK293 cells were transfected with indicated siRNA for 48 h, the mRNA levels of PSME3 and PSME4 were analyzed by RT-qPCR. Data are mean ± SEM of biological replicates (n = 3). (B) HEK293 cells transfected with indicated plasmids and siRNA for 72 h. The cells were then lysed and subjected to western blot analysis with indicated antibodies. (C) Quantitative analysis of results in (B). Data are mean ± SEM of biological replicates (n = 3). (D) Hela cells were transfected with the indicated plasmids that expressing FLAG-tagged p62 mutants. After 24 h, the cells were fixed and immunostained with anti-FLAG antibody. Nuclei were stained with DAPI (blue). Scale bar: 10 μm. (E) p62-/- cells were transfected with the indicated plasmids. After 24 h, the cells were lysed and subjected to western blot analysis with indicated antibodies. (F) Quantitative analysis of results in (E). Data are mean ± SEM of biological replicates (n = 3). (G) p62-/- cells were transfected with the indicated plasmids. After 24 h, the cells were treated with MG132 (2 μM) and LMB (2 μM) for 16 hours. After cross-linked with DTBP, the cells were lysed and used to perform immunoprecipitation using anti-Myc antibody. The results were analyzed by western blot with the indicated antibodies. (H) HEK293 cells were transfected with the indicated plasmids. After 24 h, the cells were cross-linked with DTBP, lysed and used to perform immunoprecipitation using anti-Myc antibody. The results were analyzed by western blot with the indicated antibodies. For A and C, the P value was determined by Student’s t test (two-sided). For F, the P value was determined by a one-way ANOVA analysis. (TIF) [file pgen.1011794.s004.tif]

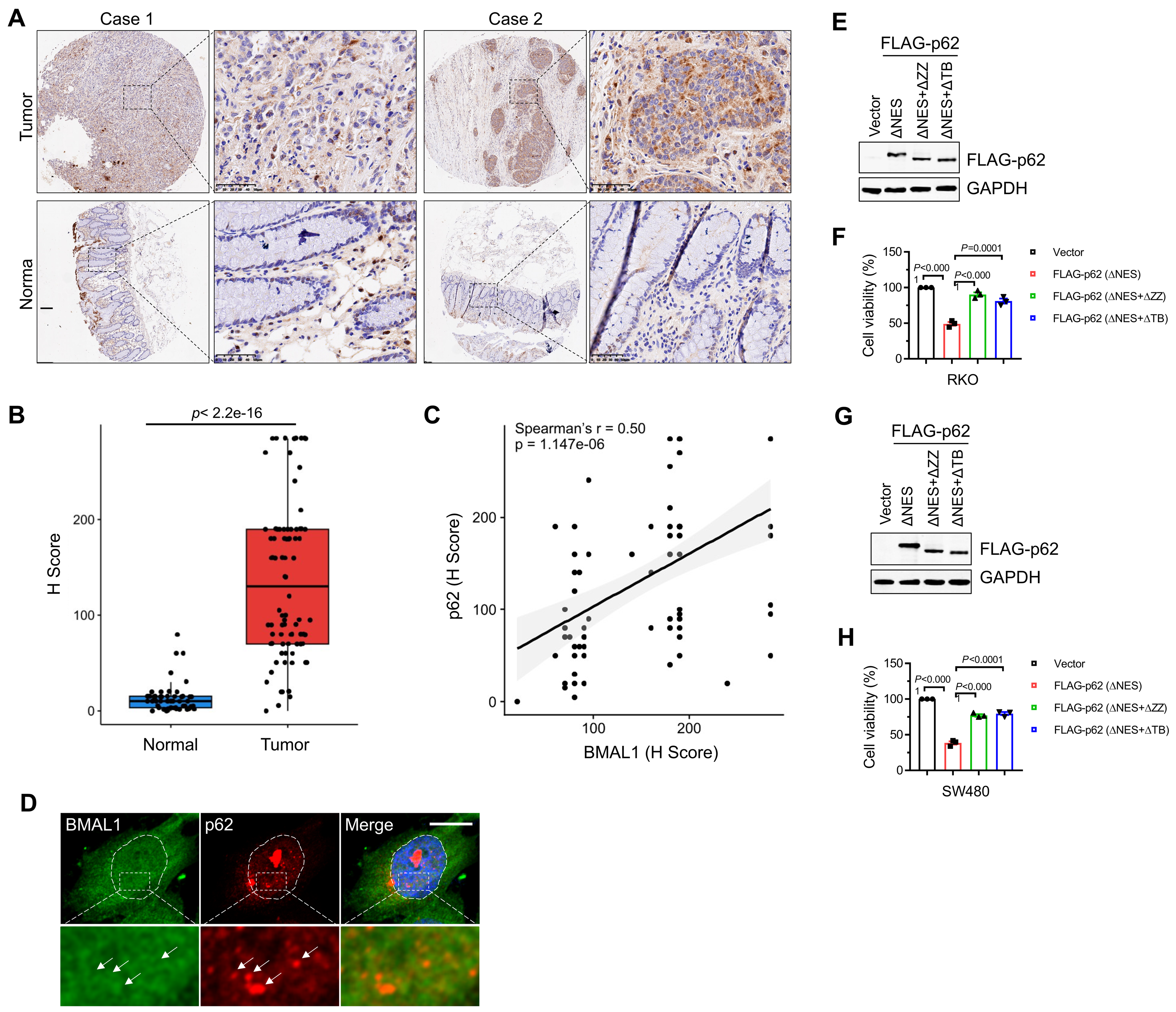

Supplement: S5 Fig — (A) Representative images of p62 expression in a tissue microarray comprising colon cancer tissues and paired normal adjacent tissues. Scale bar: 50 μm. (B) H-score values for p62 in tumor (n = 86) and normal adjacent tissues (n = 53). the P value was determined by Wilcoxon rank-sum test. (C) Spearman correlation between BMAL1 and p62 H-scores values for tumor samples (n = 86). (D) SW480 cells were treated with MG132 (2 μM) and LMB (2 μM) for 16 h. the cells were then fixed and immunostained with indicated antibodies. Nuclei were stained with DAPI (blue). Scale bar: 10 μm. (E) SW480 cells were transfected with indicated plasmids expressing mutant p62. After 48 h, the cells were lysed and subjected to western blot analysis with indicated antibodies. (F) SW480 cells were transfected with indicated plasmids expressing mutant p62. After 72 h, the cell viability was examined by CCK-8 analysis. Data are mean ± SEM of biological replicates (n = 3). (G) RKO cells were transfected with indicated plasmids expressing mutant p62. After 48 h, the cells were lysed and subjected to western blot analysis with indicated antibodies. (H) RKO cells were transfected with indicated plasmids expressing mutant p62. After 72 h, the cell viability was examined by CCK-8 analysis. Data are mean ± SEM of biological replicates (n = 3). For F and H, the P value was determined by Student’s t test (two-sided). (TIF) [file pgen.1011794.s005.tif]
